# Treatment of recurrent malignant gliomas with fotemustine monotherapy: impact of dose and correlation with MGMT promoter methylation

**DOI:** 10.1186/1471-2407-9-101

**Published:** 2009-03-31

**Authors:** Alessandra Fabi, Giulio Metro, Michelangelo Russillo, Antonello Vidiri, Carmine Maria Carapella, Marta Maschio, Francesco Cognetti, Bruno Jandolo, Maria Alessandra Mirri, Isabella Sperduti, Stefano Telera, Mariantonia Carosi, Andrea Pace

**Affiliations:** 1 Division of Medical Oncology, Regina Elena Cancer Institute, Rome, Italy; 2 Diagnostic Imaging, Regina Elena Cancer Institute, Rome, Italy; 3 Division of Neurosurgery, Regina Elena Cancer Institute, Rome, Italy; 4 Division of Neurology, Regina Elena Cancer Institute, Rome, Italy; 5 Division of Radiotherapy, Regina Elena Cancer Institute, Rome, Italy; 6 Biostatistics, Regina Elena Cancer Institute, Rome, Italy; 7 Department of Pathology Regina Elena Cancer Institute, Rome, Italy

## Abstract

**Background:**

In recurrent malignant gliomas (MGs), a high rate of haematological toxicity is observed with the use of fotemustine at the conventional schedule (100 mg/m^2 ^weekly for 3 consecutive weeks followed by triweekly administration after a 5-week rest period). Also, the impact of O6-methylguanine-DNA methyltransferase (MGMT) promoter methylation status on fotemustine activity has never been explored in the clinical setting.

**Methods:**

40 patients with recurrent pretreated MG were identified as being treated with fotemustine at doses ranging from 65 mg/m^2 ^to 100 mg/m^2^. Patients were classified into 3 groups according to the dose of fotemustine received, from the lowest dosage received in group A, to the highest in group C. Analysis of MGMT promoter methylation in tumor tissue was successfully performed in 19 patients.

**Results:**

Overall, 20% of patients responded to treatment, for a disease control rate (DCR, responses plus stabilizations) of 47.5%. Groups A and B experienced a response rate of 40% and 26.5% respectively, while the corresponding value for group C was 10%. Out of 19 patients, MGMT promoter was found methylated in 12 cases among which a DCR of 66.5% was observed. All 7 patients with unmethylated MGMT promoter were progressive to fotemustine.

**Conclusion:**

Low-dose fotemustine at 65–75 mg/m^2 ^(induction phase) followed by 75–85 mg/m^2 ^(maintenance phase) has an activity comparable to that of the conventional schedule. By determination of the MGMT promoter methylation status patients might be identified who are more likely to benefit from fotemustine chemotherapy.

## Background

Malignant gliomas (MGs) account for approximately 50% of all malignant primary brain tumors in adults [1]. Standard therapy for newly diagnosed disease includes surgical resection when feasible, radiotherapy and chemotherapy. Particularly, the role of chemotherapy has progressively become more important ever since a metanalysis suggested a small but significant increase in the 1-year survival rate of MG patients treated with adjuvant chemotherapy [2]. However, despite optimal treatment, median survival ranges from 12 to 15 months for glioblastoma multiforme (GBM) and from 2 to 5 years for anaplastic gliomas [3]. Such a dismal prognosis is mainly to ascribe to the rapid onset of radio- and/or chemo-resistance as well as to the limited therapeutic options available for MGs recurring after standard treatment.

Fotemustine is an alkylating cytotoxic agent belonging to the nitrosurea family [4]. Its elevated lipophilic properties, higher than those of other classical nitrosoureas such as carmustine (BCNU) and lomustine (CCNU), allow the drug to better penetrate through the blood-brain barrier and into malignant cells [5,6]. As single-agent, fotemustine has shown an activity ranging from 15.5% to 26% in recurrent MGs [7-9]. However, at the conventional schedule of 100 mg/m^2 ^weekly for 3 consecutive weeks followed by triweekly administration after a 5-week rest period, myelosuppression represents a considerable issue. In fact, in a phase II study by Frenay et al., 23% and 17% of all patients developed grade 3 and 4 thrombocytopenia and leukopenia respectively, with severe myelosuppression being reported in more than 30% of the subpopulation pretreated with chemotherapy [8]. More recently, even higher rates of myelotoxicity were recorded by Trevisan et al. where fotemustine monotherapy led to grade 3 and 4 thrombocytopenia and leukopenia in 55.6% and 50.6% of patients respectively [9]. The frequent development of severe haematological toxicity occurring with the conventional schedule of fotemustine might result into impairment of treatment activity due to dose omissions and/or reductions.

Preclinical evidence suggests that the O6-methylguanine-DNA methyltransferase (MGMT) repair protein is involved in resistance to alkylating agents including fotemustine [10-12]. That is because MGMT is implicated in the removal of DNA alkyl adducts from the O^6 ^position of guanine, one of the targets of alkylating drugs. Methylation of the MGMT promoter results in gene inactivation, thus potentially leading to increased sensitivity to treatment. In GBM, the MGMT promoter methylation has been proven to be a positive outcome predictor of treatment with the alkylating agent temozolomide [13]. However, no study has ever related in the clinical setting the MGMT promoter methylation status to the activity of fotemustine chemotherapy.

In order to address the importance of the dose of fotemustine in the treatment of recurrent MGs, we conducted an observational study evaluating the activity and safety of different doses of fotemustine monotherapy. In patients with available tissue the MGMT promoter methylation status was assessed.

## Methods

### Population and treatment plan

The medical records of the Regina Elena Cancer Institute in Rome were reviewed in order to identify patients with histologically proven MG (glioblastoma multiforme, anaplastic astrocytoma, anaplastic oligoastrocytoma and anaplastic oligodendroglioma) who had been treated with single-agent fotemustine as second- or third-line chemotherapy, regardless of the dose of fotemustine received. Eligible patients were required to have radiological evidence of tumor recurrence or progression prior to initiation of fotemustine chemotherapy. Moreover, to be eligible all patients had to have received at least one prior line of chemotherapy.

Retrospective chart review was approved by the Institutional Review Board of the Regina Elena Cancer Institute.

Fourty patients (table 1) were identified as being treated with i.v. fotemustine at doses ranging from 65 mg/m^2 ^to 100 mg/m^2 ^weekly for 3 consecutive cycles (induction phase) followed by a 5-week rest period, after which treatment was resumed with cycles of triweekly fotemustine at doses ranging from 75 mg/m^2 ^to 100 mg/m^2 ^(maintenance phase). Doses of fotemustine were given at physician discretion in relation to Karnofsky performance status (KPS) of each patient. For analysis purposes, patients were classified into 3 groups according to the dose of fotemustine received (from the lowest dosage received in group A, to the highest in group C) (table 2). Once a certain dosage of fotemustine was adopted, either in the induction or maintenance phase, it was never escalated in the same patient. In the absence of withdrawal of the patient or unacceptable toxicity, treatment was continued until disease progression.

**Table 1:** Patients characteristics

Characteristic	All patients no. = 40
**Median age, years (range)**	42.1 (26–76)
**Median KPS**	80 (60–100)
**Histotype:**	
Glioblastoma multiforme	14 (35%)
Anaplastic astrocytoma	11 (27.5%)
Anaplastic oligoastrocytoma	7 (17.5%)
Anaplastic oligodendroglioma	8 (20%)
**Prior surgery**	
Biopsy	8 (20%)
Partial resection	7 (17.5%)
Total resection	25 (62.5%)
**Prior radiotherapy**	40 (100%)
**Prior lines of chemotherapy**	
1	30 (75%)
2	10 (25%)
**Type of prior chemotherapy**	
TMZ	30 (75%)
PCV – TMZ	10 (25%)
**Second surgery**	19 (47.5%)
**Median time from diagnosis, months (range)**	20 (8–173)
Glioblastoma multiforme	10 (8–108)
Anaplastic astrocytoma	28.4 (10–60)
Anaplastic Oligoastrocytoma	35 (9–173)
Anaplastic Oligodendroglioma	25 (11–118)

KPS, Karnofsky Performance Status; no, number; PCV, procarbazine-lomustine-vincristine; TMZ, temozolomide

**Table 2:** Distribution of patients according to the dose of fotemustine received

Group	Induction dose, mg/m^2 ^(no. pts)	Maintenance dose, mg/m^2 ^(no. of pts)	All pts no. = 40
**A**	65 (5)	75 (5)	5
**B**	75 (15)	75 (10) – 85 (5)	15
**C**	85 (10) – 100 (10)	75/85 (18) – 100 (2)	20

no., number; pts, patients

### Response and toxicity assessment

Tumor evaluation was performed through brain MRI. Response to treatment was assessed at baseline, before the start of maintenance fotemustine and every 3 cycles thereafter or whenever disease progression was clinically suspected. Macdonald criteria were uniformly adopted for response evaluation [14].

Toxicity was assessed before each fotemustine administration by medical history, physical examination, hematology and biochemistry. Adverse events were graded 1–4 according to NCI-CTCAEv3 version 3.0 [15]. Fotemustine administration was omitted in case of grade 3–4 neutropenia and/or thrombocytopenia, febrile neutropenia and grade 3–4 non-hematological toxicity except for nausea/vomiting. At recovery treatment was resumed with a 25% dose reduction.

Serotonin antagonists were commonly administered for anti-emetic prophylaxis. Anti-epileptics were administered as medically indicated. Glucocorticoids were given to the lowest dose necessary for neurologic stability and any modification of steroidal therapy was considered when evaluating response according the criteria of Macdonald et al. [14].

### MGMT promoter methylation analysis

Genomic DNA was isolated from one paraffin section of malignant glioma tissue collected at the time of first diagnosis (Ex-Wax DNA Extraction Kit S4530, Chemicon) (proteinase digestion lasted a maximum of six hours). DNA was denatured with sodium hydroxide in a volume of 35 μl and subjected to bisulfite treatment in a volume of 350 μl (4.4 M sodium bisulfite and 20 mM hydroquinone) for five hours at 55°C and then purified. Unmethylated cytosine, but not its methylated counterpart, is modified into uracil by the treatment. The methylation-specific PCR was performed in a two-step approach. The results were confirmed in an independent experiment, starting with reisolation of DNA from the tumor. The PCR products were separated on 4 percent agarose gels.

### Statistical Analysis

Descriptive statistics were used to summarize pertinent study information. The objective response rate was reported with its 95% confidence interval. The association between variables was tested by the Pearson Chi-Square test or Fisher's Exact test. Disease control rate (DCR) was the sum of partial responses plus stable disease. Progression-free survival (PFS) and overall survival (OS) were calculated by the Kaplan-Meier product-limit method. PFS was the time elapsing from the start of fotemustine therapy to the date of objective evidence of disease progression or death of the patient in the absence of documented disease progression. OS was estimated from the first day of treatment with fotemustine to the date of death of the patient due to any cause. If a patient had not progressed/died, progression and survival were censored at the time of the last visit. The log-rank test was used to assess differences between subgroups. Significance was defined at the p < 0.05 level [16]. The Hazard risk and the confidence limits were estimated for each variable using the Cox univariate model and adopting the most suitable prognostic category as referent group [17]. A multivariate Cox proportional hazard model was also developed using stepwise regression (forward selection) with predictive variables which were significant in the univariate analyses. Enter limit and remove limit were p = 0.10 and p = 0.15 respectively. The SPSS (13.0) statistical program was used for analysis.

## Results

### Patients characteristics

The characteristics of the 40 patients are listed in table 1. Median age was 42.1 years and GBM was the most represented histotype (35% of cases). Approximately two third of patients had received total resection as primary surgery for MG and 47.5% of patients had undergone second surgery at disease recurrence. All patients had been previously treated with standard curative radiotherapy. Seventy-five percent of patients had been administered temozolomide as first-line chemotherapy, while the remainder had been treated with upfront procarbazine-lomustine-vincristine (PCV) therapy followed by second-line temozolomide. All patients completed the induction phase of fotemustine and received at least one cycle of fotemustine in the maintenance phase.

### Activity

The median number of cycles administered was 6 (range 4–8). Overall activity comprised 8 partial responses (20%; C.I.95%: 7.6–32.4) and 11 (27.5%) disease stabilizations for a DCR of 47.5%. Twenty-one patients (52.5%) experienced disease progression. All responding patients had previously responded to temozolomide chemotherapy (data not shown).

### Progression free survival and overall survival

At a median follow-up of 10 months (range 1–42), median PFS was 4 months (95% CI 2.0–5.6). The rate of patients who were free of progression at 6 and 12 months was 27% and 3.5% respectively.

**Figure 1:**
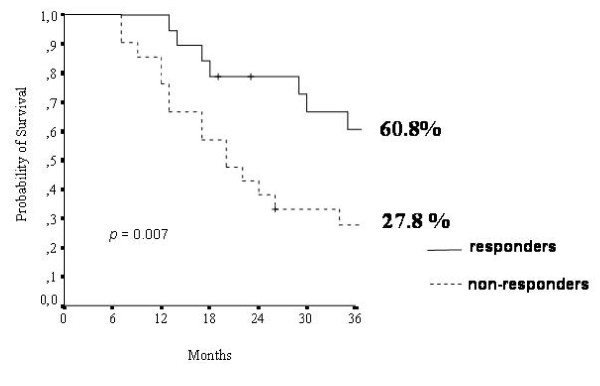
**Overall Survival in relation to response to fotemustine (all patients, n = 40)**. Responders = patients achieving partial response – Non-responders = patients achieving either stable or progressive disease.

Median OS was 30 months (95% CI 18.6–42.1). At 24 and 48 months from the start of fotemustine therapy, 87.5% and 57.5% of patients were alive respectively. OS was significantly higher among responders to fotemustine as compared to non-responders (60.8% versus 27.8%, p = 0.007) (figure 1).

### Activity according to dose, histotype and line of chemotherapy

Table 3 shows the activity of fotemustine chemotherapy according to the adopted dosage and the histotype. Groups A and B showed a response rate of 40% and 26.5% respectively, whereas patients in group C responded in 10% of cases. The median number of fotemustine cycles in groups A and B was 6 (range 6–8), while the corresponding value for group C was 4 (range 4–6). The cumulative distribution of GBM was 40%, 26.5% and 40% for groups A, B and C, respectively.

**Table 3:** Activity of fotemustine in relation to group of dose and histotype

	Group A, no. pts (%) (histotype)	Group B, no. pts (%) (histotype)	Group C, no. pts (%) (histotype)
**Partial Response**	2 (40%) *(2 AA)*	4 (26.5%) *(1 GBM, 3 AOD)*	2 (10%) *(1 AA, 1 AOD)*
**Stable Disease**	2 (40%) *(1 GBM, 1 AA)*	4 (26.5%) *(1 GBM, 2 AA, 1 AOD)*	5 (25%) *(2 GBM, 1 AA, 2 AOA)*
**Progression**	1 (20%) *(1 GBM)*	7 (47%) *(2 GBM, 2 AA, 3 AOA)*	13 (65%) *(6 GBM, 2 AA, 2 AOA, 3 AOD)*
**Total pts**	**5**	**15**	**20**
**Total GBM**	***2/5 (40%)***	***4/15 (26.5%)***	***8/20 (40%)***

AA, anaplastic astrocytoma; AOA, anaplastic oligoastrocytoma, AOD, anaplastic oligodendroglioma; GBM, glioblastoma multiforme; no., number; pts, patients

Among the 30 patients who received fotemustine as second-line treatment, DCR was 46.5%, while a DCR of 50% was observed in the 10 patients who were administered fotemustine as third-line therapy (Table 4).

**Table 4:** Activity of fotemustine according to line of chemotherapy and histotype

	Activity in 2^nd ^line no. pts (%) (histotype)	Activity in 3^rd^line no. pts (%) (histotype)	Total pts
**Partial Response**	5 (16.5%) *(1 GBM, 2 AA, 2 AOD)*	3 (30%) *(1 AA, 2 AOD)*	8
**Stable Disease**	9 (30%) (*3 GBM, 4 AA, 1 AOA, 1 AOD)*	2 (20%) *(1 GBM, 1 AOA)*	11
**Progression**	16 (53.5%) *(7 GBM, 3 AA, 4 AOA, 2 AOD)*	5 (50%) *(2 GBM, 1 AA, 1 AOA, 1 AOD)*	21
**Total pts**	**30**	**10**	**40**

AA, anaplastic astrocytoma; AOA, anaplastic oligoastrocytoma, AOD, anaplastic oligodendroglioma; GBM, glioblastoma multiforme; no., number; pts, patients

### Multivariate analysis

At the multivariate analysis, among the examined variables (age, histotype, surgery, response to first-line and fotemustine therapy) activity of fotemustine chemotherapy (DCR versus progressive disease) was an independent prognostic factor for both PFS (p < 0.0001) and OS (p = 0.02), whereas the presence of a less aggressive histotype (anaplastic oligoastrocytoma + anaplastic oligodendroglioma versus others) and activity of first-line chemotherapy (DCR versus progressive disease) were independent prognostic factors for OS (p = 0.08 and p = 0.005 respectively) (data not shown).

### Toxicity

Grade 3 and 4 thrombocytopenia and neutropenia occurred in 8 (20%) and 6 (15%) patients respectively (table 5). They were only observed in group C patients. Severe thrombocytopenia and neutropenia led to a 25% dose reduction in 8 patients in group C.

**Table 5:** Grade 3–4 toxicities per patient (all patients n = 40)

	Grade 3–4 haematologic toxicity		
Group	Neutropenia, no. pts (%)	Thrombocytopenia, no. pts (%)	Anemia, no. pts (%)
**A**	-	-	1 (2.5%)
**B**	-	-	-
**C**	6 (15%)	8 (20%)	2 (5%)

	**Grade 3–4 non-haematologic toxicity**		
**Group**	**Hepatic, n. pts (%)**	**Mucositis, n. pts (%)**	**Nausea/vomiting, n. pts (%)**

**A**	-	-	-
**B**	-	1 (2.5%)	-
**C**	2 (5%)	-	2 (5%)

Besides grade 3 hepatic toxicity and grade 3 emesis occurring in 2 patients in group C, no other severe non-hemathological toxicity were recorded (table 5).

### MGMT analysis

The assessment of the MGMT promoter methylation status was successfully performed in tumors from 19 individuals (table 6). MGMT promoter was found methylated in 12 patients, among which 3 responses and 5 disease stabilizations were observed (DCR of 66.5%). Progressive disease was recorded in all 7 patients with unmethylated MGMT.

Median PFS of patients with methylated MGMT promoter was 7 months (range 1–12) versus 6 months (range 1–10) of the unmethylated patients (p = 0.55). Median OS was 45 months (95% CI 14.0–76.5) for MGMT methylated patients versus 22 months (95% CI 17.1–27.5) for MGMT unmethylated patients (p = 0.27).

**Table 6:** Activity of fotemustine according to the MGMT promoter methylation status

	Methylated MGMT	Unmethylated MGMT	Total pts
**Partial Response**	3 (1 GBM, 2 AA)	**-**	**3**
**Stable Disease**	5 (3 GBM, 2 AA)	**-**	**5**
**Progression**	4 (2 GBM, 2 AA)	7 (4 GBM, 2 AA, 1 AOA)	**11**
**Total pts**	**12**	**7**	**19**

AA, anaplastic astrocytoma; AOA, anaplastic oligoastrocytoma, AOD, anaplastic oligodendroglioma; GBM, glioblastoma multiforme; MGMT, methylguanine methyltransferase; pts, patients

## Discussion

Medical treatment of recurrent MG is challenging. The poor availability of active chemotherapeutic drugs represents a major limitation in the decision-making process guiding treatment choice after failure of first-line chemotherapy. In addition to this, rapid tumor progression and low KPS of the patient are other important factors hampering re-treatment with chemotherapy. Against this background, recommendations for medical treatment of recurrent pretreated MGs are based almost exclusively on uncontrolled phase II studies.

In the present analysis, we found that fotemustine monotherapy is able to provide a response of 20% for an overall DCR of 47.5%, which is remarkable since these findings were observed in a heavily pretreated population (all patients had received at least one line of chemotherapy and 25% of patients had received two prior lines). These activity results are in line with those reported in the literature with single-agent fotemustine at the conventional schedule of 100 mg/m^2^, where a response rate of 15.5%–26% and a DCR of 50%–76% have been reported [7-9]. However, it should be noted that a less extensively pretreated population was included in the oldest studies (0–26.5% of patients had received previous chemotherapy) and, more importantly, CT scan instead of MRI was uniformly used to assess tumor response [7,8]. On the other hand, our PFS at 6 months of 27% compares favorably with a PFS-6 months of 15% observed in a very recent report by Trevisan et al. [9]. To this regard, it is reasonable to argue whether or not this difference in PFS should be attributed to the lower dose of fotemustine adopted in the majority of our patients which might have resulted into better tolerability of treatment. In fact, the population of the two studies is similar in that both reports include patients with recurrent MGs pretreated with ≥ 1 line of chemotherapy; yet, a much higher incidence of severe myelosuppression was recorded by Trevisan et al. with the use of a conventional schedule of fotemustine at 100 mg/m^2 ^[9].

Interestingly, in our study fotemustine monotherapy produced more enthusiastic results than those reported with "older" nitrosureas. In a retrospective study, Kappelle et al. found a response of only 3% with the classical triple combination PCV in recurrent GBM [18]. More recently, Rosenthal et al. reported 4% of responses with the use of BCNU for recurrent MG patients pretreated with temozolomide [19]. Better activity results were obtained in GBM patients with the combination of BCNU and irinotecan [20], although polychemotherapy for recurrent MGs is usually associated with higher toxicity and is bound to a strong bias of patients selection.

Notably, our analysis also showed the absence of cross-resistance between fotemustine and temozolomide, since all responses were observed in temozolomide-pretreated patients. This observation is worthy of being pointed out in view of the recent incorporation of temozolomide in the standard treatment of newly diagnosed glioblastoma multiforme [21]. Similarly, other authors have reported an activity of 30% with the use of fotemustine in glioblastoma patients pretreated with temozolomide [22]. On the other hand, the lack of activity observed for fotemustine in PCV-pretreated patients, suggests the presence of cross-resistance between fotemustine and other nitrosureas as hypothesized preclinically [23].

Furthermore, our results showed that both the activity of first-line therapy and treatment with fotemustine were positive prognostic factors for OS; these findings confirm that the use of chemotherapy in recurrent MGs has a positive impact on patients outcome [24].

The better activity recorded in groups A and B where fotemustine was given at doses ranging from 65 mg/m^2 ^to 85 mg/m^2 ^is supposedly to ascribe to the absence of severe myelotoxicity that has allowed the administration of a higher dose intensity. In fact, at these doses we found no case of severe thrombocytopenia and/or neutropenia, whereas the same adverse events were 20% and 15% respectively in group C, where fotemustine was given at doses ranging from 75–85 mg/m^2 ^to 100 mg/m^2^. Importantly, our data appear to rule out the hypothesis that the better activity reported for low-dose fotemustine could be attributed to an imbalanced distribution toward group A and B of tumors with a worse prognosis such as GBM (table 3).

Interestingly, fotemustine-induced myelosuppression can also be lowered by delaying the intervals between each cycle, as shown recently in two studies exploring fotemustine in combination with either dacarbazine or procarbazine for the treatment of recurrent GBM [25,26]. However, the modest response (3%–11%) observed in these studies showed also that the use of a fotemustine schedule not including the weekly induction phase might compromise the activity of fotemustine itself through a higher rate of early progressions, thus invalidating the benefits potentially obtainable by the addition of a second cytotoxic [25,26]. To this regard, in our study no cases of early progression were recorded after the induction phase and all patients received at least six cycles of fotemustine.

In the 19 patients with tissue available for assessment of the MGMT promoter methylation status, we found a considerable rate of disease control in patients with methylated MGMT (8 out of 12, 66.5%) (table 6). More interestingly, all of the 7 unmethylated patients were found progressive to fotemustine monotherapy. Despite the low number of patients analyzed and the heterogeneity of patients histotypes, these data suggest that the presence of MGMT promoter methylation is a crucial prerequisite for response to fotemustine although it does not guarantee sensitivity to treatment. On the other hand, patients with unmethylated MGMT do not appear to benefit at all from fotemustine chemotherapy. However, our number of patients was too low to observe a significant difference in terms of efficacy according to MGMT promoter methylation status. For this reason, the role of MGMT should be further assessed prospectively in a larger cohort of patients undergoing fotemustine chemotherapy, possibly re-assessing MGMT status at the time of tumor recurrence. In fact, a recent study suggested that changes in the status of MGMT promoter methylation may occur after primary treatment for newly diagnosed GBM [27].

## Conclusions

This study provides a solid rationale for testing low-dose fotemustine in the treatment of recurrent MGs. On this basis, a phase II study investigating fotemustine at the induction dose of 65 mg/m^2 ^followed by a maintenance dose of 75 mg/m^2 ^is currently ongoing at 5 different Italian institutions. Importantly, prospective evaluation of MGMT methylation is mandatory in patients with tissue availability.

## Competing interests

The authors declare that they have no competing interests.

## Authors' contributions

AF and AP were responsible for the conception of the study. AF, GM, MR and AP were responsible for the assembly and analysis/interpretation of data. GM drafted the manuscript. IS did the statistical analysis. MC performed the MGMT essay. AF, AV, CMC, MM, FC, BJ, MAM, ST and AP provided study patients. All authors revised the manuscript critically and gave their approval for it to be published in its final version.

## Pre-publication history

The pre-publication history for this paper can be accessed here:

http://www.biomedcentral.com/1471-2407/9/101/prepub

## References

[B1] De Vita VT Hellman S Rosenberg SA Cancer: principles & practice of oncology 2008 8 Philadelphia: Lippincott Williams & Wilkins

[B2] Stewart LA Chemotherapy in adult high-grade glioma: a systematic review and meta-analysis of individual patient data from 12 randomised trials Lancet 2002 359 1011 1018 10.1016/S0140-6736(02)08091-1 11937180

[B3] Wen PY Kesari S Malignant gliomas in adults N Engl J Med 2008 359 492 507 10.1056/NEJMra0708126 18669428

[B4] Hayes MT Bartley J Parsons PG Eaglesham GK Prakash AS Mechanism of action of fotemustine, a new chloroethylnitrosourea anticancer agent: evidence for the formation of two DNA-reactive intermediates contributing to cytotoxicity Biochemistry 1997 36 10646 10654 10.1021/bi970791q 9271495

[B5] Levin VA Relationship of octanol/water partition coefficient and molecular weight to rat brain capillary permeability Med Chem 1980 23 682 684 10.1021/jm00180a022 7392035

[B6] Meulemans A Giroux B Hannoun P Robine D Henzel D Comparative diffusion study of two nitrosoureas: carmustine and fotemustine in normal rat brain, human and rat brain biopsies Chemotherapy 1991 37 86 92 2032474 10.1159/000238838

[B7] Malhaire JP Lucas B Simon H Person H Dam-Hieu P Labat JP Fotemustine (Muphoran) in 22 patients with relapses of high-grade cerebral gliomas Bull Cancer 1999 86 289 294 10210763

[B8] Frenay M Giroux B Khoury S Derlon JM Namer M Phase II study of fotemustine in recurrent supratentorial malignant gliomas Eur J Cancer 1991 27 852 856 1834116 10.1016/0277-5379(91)90133-x

[B9] Trevisan E Laguzzi E Ruda R Guarneri D Soffietti R Safety and efficacy of fotemustine in recurrent or progressive gliomas [abstract] J Neurol 2008 255 s93 10.1007/s00415-008-6017-7

[B10] Esteller M Herman JG Generating mutations but providing chemosensitivity: the role of O6-methylguanine DNA methyltransferase in human cancer Oncogene 2004 23 1 8 10.1038/sj.onc.1207316 14712205

[B11] Kaina B Mühlhausen U Piee-Staffa A Christmann M Garcia Boy R Rösch F Schirrmacher R Inhibition of O6-methylguanine-DNA methyltransferase by glucose-conjugated inhibitors: comparison with nonconjugated inhibitors and effect on fotemustine and temozolomide-induced cell death J Pharmacol Exp Ther 2004 311 585 593 10.1124/jpet.104.071316 15254145

[B12] Kaina B Christmann M Naumann S Roos WP MGMT: key node in the battle against genotoxicity, carcinogenicity and apoptosis induced by alkylating agents DNA Repair (Amst) 2007 6 1079 1099 10.1016/j.dnarep.2007.03.008 17485253

[B13] Hegi ME Diserens AC Gorlia T Hamou MF de Tribolet N Weller M Kros JM Hainfellner JA Mason W Mariani L Bromberg JE Hau P Mirimanoff RO Cairncross JG Janzer RC Stupp R MGMT gene silencing and benefit from temozolomide in glioblastoma N Engl J Med 2005 352 997 1003 10.1056/NEJMoa043331 15758010

[B14] Macdonald DR Cascino TL Schold SC Jr Cairncross JG Response criteria for phase II studies of supratentorial malignant glioma J Clin Oncol 1990 8 1277 1280 2358840 10.1200/JCO.1990.8.7.1277

[B15] Common Terminology Criteria for Adverse Events v3.0 (CTCAE) http://ctep.cancer.gov/protocolDevelopment/electronic_applications/ctc.htm

[B16] Kaplan EL Meier P Non parametric estimation from incomplete observations J Am Stat Assoc 1958 53 457 481 10.2307/2281868

[B17] Cox DR Regression models and life tables J Royal Stat Soc 1972 4 187 200

[B18] Kappelle AC Postma TJ Taphoorn MJ Groeneveld GJ Bent MJ van den van Groeningen CJ Zonnenberg BA Sneeuw KC Heimans JJ PCV chemotherapy for recurrent glioblastoma multiforme Neurology 2001 56 118 120 11148250 10.1212/wnl.56.1.118

[B19] Rosenthal MA Ashley DL Cher L BCNU as second line therapy for recurrent high-grade glioma previously treated with Temozolomide J Clin Neurosci 2004 11 374 375 10.1016/j.jocn.2003.01.002 15080950

[B20] Brandes AA Tosoni A Basso U Reni M Valduga F Monfardini S Amistà P Nicolardi L Sotti G Ermani M Second-line chemotherapy with irinotecan plus carmustine in glioblastoma recurrent or progressive after first-line temozolomide chemotherapy: a phase II study of the Gruppo Italiano Cooperativo di Neuro-Oncologia (GICNO) J Clin Oncol 2004 22 4779 4786 10.1200/JCO.2004.06.181 15570079

[B21] Stupp R Mason WP Bent MJ van den Weller M Fisher B Taphoorn MJ Belanger K Brandes AA Marosi C Bogdahn U Curschmann J Janzer RC Ludwin SK Gorlia T Allgeier A Lacombe D Cairncross JG Eisenhauer E Mirimanoff RO Radiotherapy plus concomitant and adjuvant temozolomide for glioblastoma N Engl J Med 2005 352 987 996 10.1056/NEJMoa043330 15758009

[B22] Scoccianti S Detti B Sardaro A Iannalfi A Meattini I Leonulli BG Borghesi S Martinelli F Bordi L Ammannati F Biti G Second-line chemotherapy with fotemustine in temozolomide-pretreated patients with relapsing glioblastoma: a single institution experience Anticancer Drugs 2008 19 613 620 10.1097/CAD.0b013e3283005075 18525321

[B23] Filippeschi S Colombo T Bassani D De Francesco L Arioli P D'Incalci M Bartosek I Guaitani A Antitumor activity of the novel nitrosourea S10036 in rodent tumors Anticancer Res 1988 8 1351 1354 3064716

[B24] Filippini G Falcone C Boiardi A Broggi G Bruzzone MG Caldiroli D Farina R Farinotti M Fariselli L Finocchiaro G Giombini S Pollo B Savoiardo M Solero CL Valsecchi MG Prognostic factors for survival in 676 consecutive patients with newly diagnosed primary glioblastoma Neuro Oncol 2008 10 79 87 17993634 10.1215/15228517-2007-038 PMC2600841

[B25] Fazeny-Dörner B Veitl M Wenzel C Piribauer M Rössler K Dieckmann K Ungersböck K Marosi C Second-line chemotherapy with dacarbazine and fotemustine in nitrosourea-pretreated patients with recurrent glioblastoma multiforme Anticancer Drugs 2003 14 437 442 10.1097/00001813-200307000-00008 12853885

[B26] Silvani A Lamperti E Gaviani P Eoli M Fiumani A Salmaggi A Falcone C Filippini G Botturi A Boiardi A Salvage chemotherapy with procarbazine and fotemustine combination in the treatment of temozolomide treated recurrent glioblastoma patients J Neurooncol 2008 87 143 151 10.1007/s11060-007-9427-y 17576523

[B27] Parkinson JF Wheeler HR Clarkson A McKenzie CA Biggs MT Little NS Cook RJ Messina M Robinson BG McDonald KL Variation of O(6)-methylguanine-DNA methyltransferase (MGMT) promoter methylation in serial samples in glioblastoma J Neurooncol 2008 87 71 78 10.1007/s11060-007-9486-0 18004504

